# Consensus molecular subtype classification of colorectal adenomas

**DOI:** 10.1002/path.5129

**Published:** 2018-08-31

**Authors:** Malgorzata A Komor, Linda JW Bosch, Gergana Bounova, Anne S Bolijn, Pien M Delis‐van Diemen, Christian Rausch, Youri Hoogstrate, Andrew P Stubbs, Mark de Jong, Guido Jenster, Nicole CT van Grieken, Beatriz Carvalho, Lodewyk FA Wessels, Connie R Jimenez, Remond JA Fijneman, Gerrit A Meijer, In collaboration with the NGS‐ProToCol Consortium:, Natasja Dits, Rene Bottcher, Annemieke C Hiemstra, Bauke Ylstra, Daoud Sie, Evert van den Broek, David van der Meer, Floor Pepers, Eric Caldenhoven, Bart Janssen, Wilbert van Workum, Stef van Lieshout, Chris H. Bangma, Geert van Leenders, Harmen van de Werken

**Affiliations:** ^1^ Translational Gastrointestinal Oncology, Department of Pathology Netherlands Cancer Institute Amsterdam The Netherlands; ^2^ Oncoproteomics Laboratory, Department of Medical Oncology VU University Medical Centre Amsterdam The Netherlands; ^3^ Division of Molecular Carcinogenesis The Netherlands Cancer Institute Amsterdam The Netherlands; ^4^ Department of Urology Erasmus Medical Centre Rotterdam Rotterdam The Netherlands; ^5^ Department of Bioinformatics Erasmus Medical Centre Rotterdam Rotterdam The Netherlands; ^6^ GenomeScan Leiden The Netherlands; ^7^ Department of Pathology VU University Medical Centre Amsterdam The Netherlands; ^8^ Department of Electrical Engineering, Mathematics and Computer Science Delft University of Technology Delft The Netherlands; ^9^ Lygature, Utrecht The Netherlands; ^10^ Department of Pathology, Erasmus Medical Centre Rotterdam Rotterdam The Netherlands

**Keywords:** colon, rectum, neoplasia, adenoma, colorectal cancer

## Abstract

Consensus molecular subtyping is an RNA expression‐based classification system for colorectal cancer (CRC). Genomic alterations accumulate during CRC pathogenesis, including the premalignant adenoma stage, leading to changes in RNA expression. Only a minority of adenomas progress to malignancies, a transition that is associated with specific DNA copy number aberrations or microsatellite instability (MSI). We aimed to investigate whether colorectal adenomas can already be stratified into consensus molecular subtype (CMS) classes, and whether specific CMS classes are related to the presence of specific DNA copy number aberrations associated with progression to malignancy. RNA sequencing was performed on 62 adenomas and 59 CRCs. MSI status was determined with polymerase chain reaction‐based methodology. DNA copy number was assessed by low‐coverage DNA sequencing (*n* = 30) or array‐comparative genomic hybridisation (*n* = 32). Adenomas were classified into CMS classes together with CRCs from the study cohort and from The Cancer Genome Atlas (*n* = 556), by use of the established CMS classifier. As a result, 54 of 62 (87%) adenomas were classified according to the CMS. The CMS3 ‘metabolic subtype’, which was least common among CRCs, was most prevalent among adenomas (*n* = 45; 73%). One of the two adenomas showing MSI was classified as CMS1 (2%), the ‘MSI immune’ subtype. Eight adenomas (13%) were classified as the ‘canonical’ CMS2. No adenomas were classified as the ‘mesenchymal’ CMS4, consistent with the fact that adenomas lack invasion‐associated stroma. The distribution of the CMS classes among adenomas was confirmed in an independent series. CMS3 was enriched with adenomas at low risk of progressing to CRC, whereas relatively more high‐risk adenomas were observed in CMS2. We conclude that adenomas can be stratified into the CMS classes. Considering that CMS1 and CMS2 expression signatures may mark adenomas at increased risk of progression, the distribution of the CMS classes among adenomas is consistent with the proportion of adenomas expected to progress to CRC. © 2018 The Authors. *The Journal of Pathology* published by John Wiley & Sons Ltd on behalf of Pathological Society of Great Britain and Ireland.

## Introduction

Colorectal cancer (CRC) is heterogeneous in its molecular characteristics and its treatment response. Stratifying CRC patients into biologically and clinically distinct subtypes, based on gene expression profiles, has been performed in many studies, with the common aim of improving clinical precision 1, 2, 3, 4, 5, 6, 7. Recently, a large effort was made by the CRC Subtyping Consortium to reconcile the differences between the multiple existing classifications and to derive consensus molecular subtypes (CMSs) of CRC 8. A consensus RNA expression‐based classifier was produced that classifies CRCs into four CMS groups. CMS1 includes ∼14% of CRCs, and is associated with microsatellite instability (MSI), *BRAF* mutation, promoter hypermethylation, and immune infiltration. Chromosomal instability (CIN), the most common type of genomic instability in CRC, is a feature characteristic of CMS2–CMS4. CMS2 is the most prevalent CRC subtype (37%) and shows the hallmarks of canonical CRC carcinogenesis, including activation of the Wnt and Myc pathways. Approximately 13% of CRCs are in CMS3, characterised by dysregulated metabolism and *KRAS* mutation. Finally, CMS4 (23%) is described as a mesenchymal, stroma‐rich group, associated with poor prognosis 8.

Most CRCs progress from normal epithelium, through a benign precursor adenoma, by accumulating genetic alterations in oncogenes and tumour suppressor genes 9. However, adenomas are much commoner in the large intestine than cancers, and it is estimated that only 5% eventually progress to cancer 10. Although it is evident that CMS signatures can be discerned at the CRC stage, the question remains of whether this would already be possible at the adenoma stage, and, if so, how the distribution of CMS classes would compare with that of CRCs.

A further question is whether adenomas with a high risk of progressing to cancer would differ in their CMS pattern from adenomas with a low risk of progression. In general, the progression of dysplastic epithelial premalignant lesions such as colorectal adenomas is associated with the acquisition of genomic instability. Often, this concerns aneuploidy or CIN, which marks ∼85% of CRC cases 11. CIN has been studied in CRC and its precursor lesions to identify non‐random chromosomal aberrations and potential CRC driver events. In multiple studies, a distinct pattern has been observed in colorectal lesions with CIN, which has been shown to play a major role in adenoma‐to‐carcinoma progression 12, 13, 14, 15, 16, 17, 18, 19, 20, 21. Seven copy number aberrations have been identified as colorectal cancer‐associated events (CAEs): gains of chromosomal arms 8q, 13q, and 20q, and losses of 8p, 15q, 17p, and 18q 12. With an accuracy of 78%, adenomas with at least two of the seven CAEs can be identified as being at a high risk of progressing to malignancy; these are referred to as ‘high‐risk adenomas’ 12. Integration of these DNA copy number aberrations and RNA expression data led to the identification of putative oncogenes located in the amplified regions 22, 23. Functional studies of candidate oncogenes from the 20q region indicated that *AURKA* and *TPX2* promote 20q amplicon‐driven adenoma‐to‐carcinoma progression 16. This means that the non‐random DNA copy number aberrations do, in fact, influence biological processes within cells, through which they facilitate colorectal tumourigenesis. The fact that these aberrations are present in some of the adenomas shows that the signal of malignant transformation can already be detected at a molecular level at the adenoma stage. This implies that gene expression profiles of colorectal adenomas may also carry information on the future CMS.

The present study therefore aimed to investigate whether the differentiation of colorectal epithelial neoplasia into CMS classes can already be recognised at the adenoma stage, and whether specific CMS classes are associated with the absence or presence of specific DNA copy number aberrations in colorectal adenomas that reflect a high risk of progressing to cancer.

## Materials and methods

### Sample collection

A total of 62 snap‐frozen advanced adenomas and 59 CRCs were collected from two independent sample collections: Series 1 and Series 2 (described in supplementary material, Supplementary materials and methods). Clinical information is shown in Table 1. The collection, storage and use of tissue and patient data were performed in compliance with the Code for Proper Secondary Use of Human Tissue in the Netherlands 24.

**Table 1:** Characteristics of sample Series 1 and Series 2 collected for this study

Characteristics		Number of samples		
		Series 1	Series 2	Total
Lesion	Adenoma	30	32	62
Histological type	Tubular	6	13	19
	Tubulovillous	20	16	36
	Villous	4	3	7
Dysplasia	High grade	10	8	18
	Low grade	20	24	44
Risk of progression	High	9	4	13
	Low	17	22	39
	No information	2	6	8
Microsatellite status	MSS	28	32	60
	MSI	2	0	2
Lesion	Carcinoma	30	29	59
Differentiation grade	Less/Not	4	2	6
	Well differentiated/ moderately differentiated	25	27	52
	No information	1	0	1
Stage	I	7	9	16
	II	13	10	23
	III	6	9	15
	IV	3	1	4
	I or III	1	0	1
Microsatellite status	MSS	24	23	47
	MSI	6	6	12

MSS, microsatellite‐stable.

### DNA copy number analysis

For Series 1, copy number analysis by low‐coverage whole genome sequencing was performed (supplementary material, Supplementary materials and methods and Table S1). Gains and losses of whole chromosomal arms were used for the identification of high‐risk adenomas. Samples were considered to have undetermined risk when the copy number aberrations were present but did not reach the probability cut‐off of 0.5 (*n* = 2). For Series 2, DNA copy number data for 28 adenomas were obtained from the array‐comparative genomic hybridisation (arrayCGH) analysis in an earlier study 22. Samples were considered to have undetermined risk if the arrayCGH data were unavailable (*n* = 4) or only a minor part of the chromosomal arm was gained or lost (*n* = 2). For both series, adenomas with at least two of seven CAEs were labelled as high‐risk 12.

### MSI assay

Adenoma and carcinoma samples from both series were analysed for MSI with the MSI Multiplex System Version 1.2 (Promega, Madison, WI, USA; cat. no. MD1641) according to standard procedures, as described previously 25.

### RNA sequencing (RNA‐seq) and data preprocessing

Both series were subjected to RNA‐seq and data preprocessing separately. Expression matrices were obtained for each series (supplementary material, Supplementary materials and methods and Table S1).

### Batch effect removal with respect to The Cancer Genome Atlas (TCGA) CRC data

TCGA data served as a reference for performance of the analysis in the present study 15. Expression values of 556 TCGA samples used in the original CMS classification were used for RNA‐seq data normalisation and CMS classification (supplementary material, Supplementary materials and methods).

**Figure 1:**
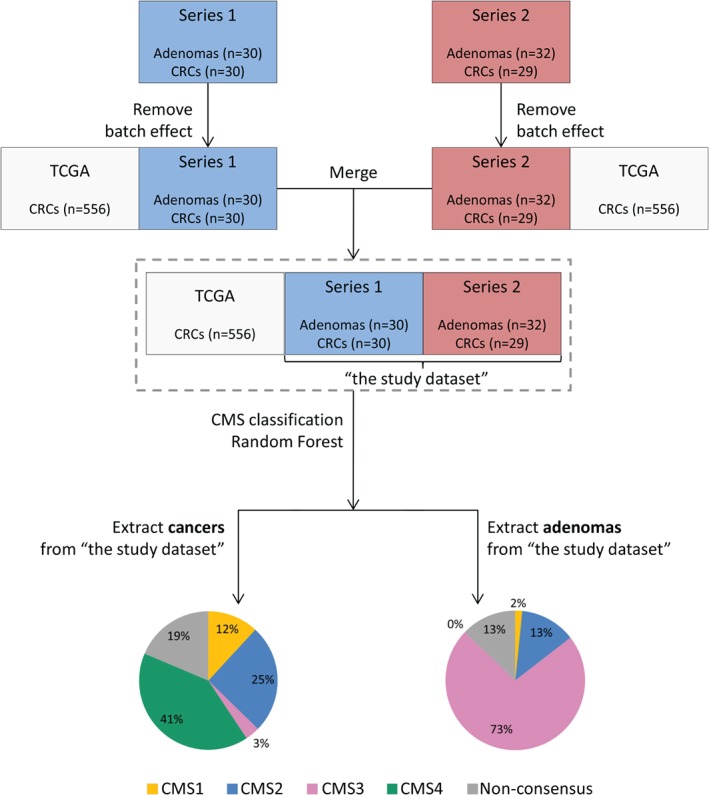
Overview of the data analysis approach. Both Series 1 and Series 2 were normalised separately to the TCGA CRC dataset via a batch effect removal method 27. After normalisation, all three datasets were merged together. Series 1 and Series 2 form the ‘study dataset’. CMS classification was applied to the merged dataset. The classes were obtained with the CMS random forest classifier, and assigned when the posterior probability of belonging to a CMS class was ≥0.5. Results of the classification were extracted for the CRCs and the adenomas from the study dataset. The pie charts represent the distribution of CMS classes for CRCs (left) and adenomas (right) for the study dataset.

The batch effect was removed with M‐Combat 26, separately for Series 1 and Series 2. In both cases, the TCGA dataset served as the reference, and Series 1 or Series 2 served as the normalised batch (Figure 1). Adenomas and cancers were kept together during the normalisation to avoid removal of the ‘lesion‐based’ variance. TCGA data as the gold‐standard reference dataset remained unchanged. All three datasets (Series 1, Series 2, and TCGA) were merged, and Series 1 and Series 2 formed the study dataset. Batch effect removal was evaluated by use of a multidimensional scaling algorithm on the Euclidian distance between the expression profiles of the samples. Evaluation of the preservation of the difference between adenomas and carcinomas was performed by the use of hierarchical clustering with complete linkage on the log_2_‐transformed RPKMs of the top 30 and the top 1000 variable genes.

### CMS classification

Ensembl IDs were translated to Entrez IDs with the biomaRt Bioconductor package 27. The random forest CMS classifier 8 was applied on the merged dataset, including TCGA dataset, Series 1, and Series 2, and a CMS class was assigned when the posterior probability of a sample belonging to a subtype was ≥0.5. To obtain the original CMS labels for TCGA samples, the random forest CMS classifier was also applied to the whole CMS dataset downloaded from the CRC Subtyping Consortium Synapse website 8, 28. CMS labels for TCGA samples were extracted. To evaluate the results of the random forest CMS classifier, the single sample predictor (SSP) classification method 8 was applied to the adenomas from Series 1 and Series 2 before normalisation to the TCGA dataset. A CMS class was assigned according to the default settings (minCor = 0.15, minDelta = 0.06).

### Validation set

To validate the results in an independent series of adenomas measured with a different platform, expression data from the Affymetrix Human Genome U133 Plus 2.0 Array of 45 colorectal adenomas and 36 CRCs (GSE20916) were downloaded from the Gene Expression Omnibus. This validation set will be referred as ‘Series 3’ 29. The reference dataset chosen was the largest series of CRCs measured with the same methodology and used in the original CMS classification (GSE39582) 3, 8. See supplementary material, Supplementary materials and methods for details of data analysis and the CMS classification of Series 3.

### Statistical analysis

The multinomial exact test was used to perform a goodness‐of‐fit test for the distributions of CMS classes in the adenomas in comparison with cancers from the study dataset, adenomas from the validation set, and cancers from the original CMS publication 8. Contingency tables including adenomas classified as CMS2 and CMS3 were analysed; CMS1 and CMS4 were excluded because of the limited number of cases. Associations analysed were clinical features, risk of progression or occurrence of each of the seven CAEs separately. A relationship was considered to be significant if the *P* value was ≤0.05 (Fisher's exact test). Additionally, associations between CMS classes in CRCs and clinical features were analysed.

### Gene set enrichment analysis (GSEA)

Prior to GSEA 30, an expression matrix after normalisation was extracted for CMS2 and CMS3 adenomas. Exponentiation with base 2 was applied, and values were rounded to integers to create count data. Differential gene expression analysis was performed with the Bioconductor package DESeq2 31, and genes were sorted on the basis of log_2_ fold change, whereby genes upregulated in CMS2 adenomas were at the top of the list. (Fold change is defined as the ratio of test to reference expression level.) The log_2_ fold change‐based ranked list was submitted to GSEA 30, and the collection of hallmark gene sets from Molecular Signature Database v6.0 was used 32. Significant gene sets were extracted on the basis of a false discovery rate (FDR) threshold of ≤0.2. For the comparison of stroma and invasion signatures between adenomas and cancers, the ESTIMATE algorithm 33 was used, as well as single‐sample GSEA with the GSVA Bioconductor package 34, with the ‘invasive front’ and ‘central tumour’ signatures 35.

## Results

### CMS classification of the cancers and the adenomas

**Figure 2:**
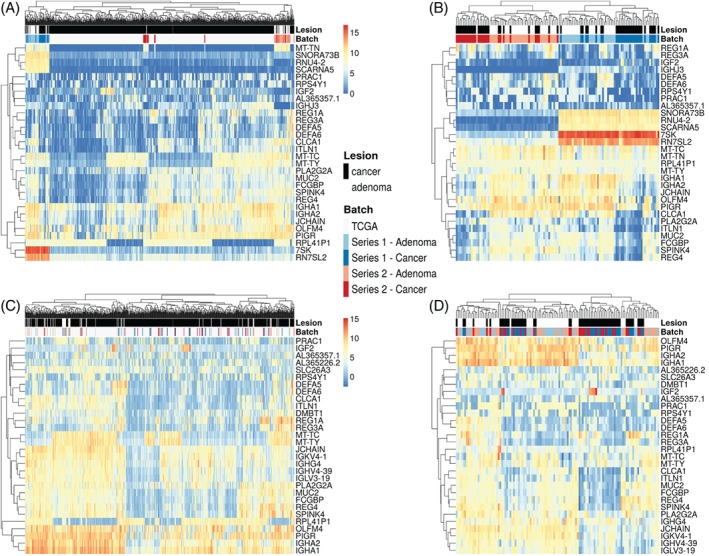
Hierarchical clustering based on gene expression profiles of the top 30 most variable genes. (A) Heatmap of all three datasets before batch effect removal. The batches corresponding to the TCGA dataset, Series 1 and Series 2 can be distinguished in the heatmap. (B) Heatmap before batch correction of the Series 1 and Series 2 study datasets only. Within the two batches, one can distinguish clusters enriched with adenomas and clusters enriched with cancers. (C) Heatmap of all three datasets after batch effect removal. Samples from the three experiments do not cluster together. (D) Heatmap of the Series 1 and Series 2 study datasets after batch effect removal. Clusters enriched with adenomas or cancers can still be distinguished, meaning that batch effect correction did not remove the variability between different lesions. The legend corresponds to all of the heatmaps in this figure.

An overview of the data analysis is shown in Figure 1. Series 1, Series 2 and the TCGA dataset originated from different experiments, representing three separate batches that needed to be normalised (supplementary material, Figure S1A). To avoid a change in the original TCGA classification, the TCGA dataset remained unchanged and was used as a gold‐standard reference for batch effect removal. Both Series 1 and Series 2 were successfully normalised to the TCGA dataset ( supplementary material, Figure S1B). Hierarchical clustering based on expression values of the top 30 and top 1000 variable genes before and after batch effect removal showed that the normalisation did not remove the differences between the adenomas and the cancers, as the lesions could still be distinguished on the basis of their expression profiles (Figure 2; supplementary material, Figure S2). The variability between cancers and adenomas was thus preserved after batch effect removal.

On the basis of two tissue datasets, Series 1 and Series 2, we collected a cohort of 62 adenomas and 59 CRCs, referred to as the study dataset. To ensure proper classification of the adenomas, which constitute a different entity from CRCs, the CMS classification was applied to a merged dataset with carcinomas from the present study (*n* = 59) and TCGA data (*n* = 556); see Figure 1 for an overview of the data analysis approach. To evaluate whether the data analysis approach had an impact on the classification, the CMS labels obtained in this study for TCGA samples were compared with their original CMS labels 8. The CMS labels of TCGA samples were reassigned in this study with an accuracy of 97%, corresponding to the previously reported overall accuracy of the random forest CMS classifier of 96% (supplementary material, Table S2) 8.

The CMS classification results of the study dataset were extracted. In total, 48 of 59 cancers were classified with a posterior probability of ≥0.5. Of these, seven were classified as CMS1, 15 as CMS2, two as CMS3, and 24 as CMS4 (Figure 1; Table 2; supplementary material, Table S3). Hence, the CMS4 mesenchymal subtype was the most prevalent in this dataset. Of the 12 samples of CRC with MSI, four were classified as CMS1, four were classified as CMS4, one was classified as CMS3, and three were not classified. Statistically significant associations of CMS classes with MSI status (*p* = 0.004) and with differentiation grade (*p* = 0.006) were observed, but no association with stage was identified (*p* = 0.235; see supplementary material, Table S4, for MSI status and association analysis).

**Table 2:** Distribution of the CMS classes in cancers and adenomas from the study dataset and the validation set

	CMS1, *n* (%)	CMS2, *n* (%)	CMS3, *n* (%)	CMS4, *n* (%)	Non‐consensus, *n* (%)
Study dataset (Series 1 and Series 2)					
Cancers	7 (12)	15 (25)	2 (3)	24 (41)	11 (19)
Adenomas	1 (2)	8 (13)	45 (73)	0 (0)	8 (13)
Validation set (Series 3)					
Cancers	5 (14)	7 (19)	1 (3)	18 (50)	5 (14)
Adenomas	1 (2)	5 (11)	28 (62)	0 (0)	11 (24)

CMS subtype signatures were indeed expressed in the adenomas, and 54 of 62 samples were successfully classified with a probability threshold of ≥0.5. The vast majority of the adenomas, i.e. 45 samples (73%), were assigned to CMS3. Additionally, eight adenomas (13%) were subtyped as CMS2, representing the canonical CRC carcinogenesis. Only a single adenoma was classified as CMS1, being one of the two MSI adenomas identified in the whole dataset. No adenomas were subtyped as CMS4 (Table 2; supplementary material, Table S5). The distribution of CMS classes in the adenomas differed significantly from that in the CRCs from the study dataset (*p* < 2.2 × 10^–16^) and CRCs from the original CMS publication (*p* < 2.2 × 10^–16^) 8.

### CMS classification of adenomas, risk of progression, and biological characterisation

Adenomas from the study dataset were called high risk on the basis of the presence of at least two of seven specific DNA copy number aberrations: 8q, 13q and 20q gains, and 8p, 15q, 17p and 18q losses 12. Adenomas with MSI were excluded, because a different genome instability process (i.e. not CIN) is involved. In total, 13 adenomas were called high risk and 39 were called low risk (Table 1; supplementary material, Table S6). No final calls could be made for the remaining eight.

**Figure 3:**
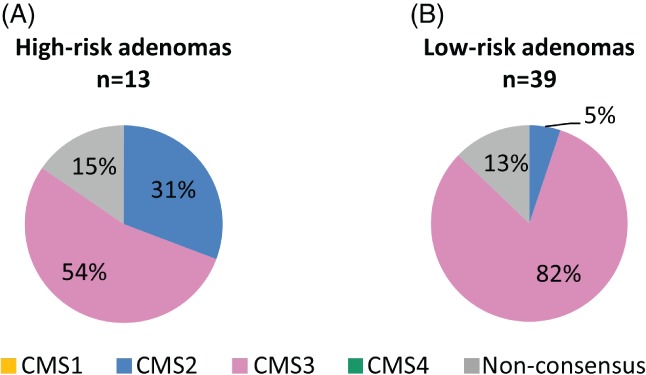
Distribution of CMS classes among adenomas at high risk and adenomas at low risk of progressing to cancer. (A) Distribution of CMS classes among 13 high‐risk adenomas. (B) Distribution of CMS classes among 39 low‐risk adenomas. No high‐risk and low‐risk adenomas were classified as CMS1 or CMS4.

Adenomas classified as CMS2 (*n* = 8) and CMS3 (*n* = 45) were the most prevalent; there were no CMS4 adenomas, and there was one adenoma classified as CMS1. Therefore, only differences between CMS2 and CMS3 adenomas were examined in terms of risk of progression, cancer‐specific DNA copy number aberrations, clinical characteristics, and biological processes specific for each group. Examination of associations between CMS class and risk of progression revealed that CMS2 was significantly associated with high‐risk adenomas and CMS3 with low‐risk adenomas (*p* = 0.025; Figure 3). When each of the seven CAEs were examined, gain of 20q and loss of 18q were significantly associated with CMS2 (*p* = 0.004 and *p* = 0.031, respectively). No statistically significant associations were observed between CMS class and histological type (*p* = 0.362) and grade of dysplasia (*p* = 0.389), or between high‐risk genotypic features and histological type (*p* = 0.77) and grade of dysplasia (*p* = 0.079; supplementary material, Table S7).

To explore associations of CMS2 and CMS3 adenomas with well‐defined biological processes, we performed GSEA on the hallmark gene sets (Table 3) 30. As expected, the gene sets enriched in CMS2 adenomas were involved in cell cycle and proliferation, including genes that are targets of E2F transcription factors, genes involved in G_2_/M checkpoint, mitotic spindle assembly, the phosphoinositide 3‐kinase (PI3K)–AKT–mammalian target of rapamycin (mTOR) pathway, and the Wnt–β‐catenin signalling pathway, or genes regulated by MYC. These results are in line with the biological characterisation of CMS2 CRCs, which are known to be enriched with proliferation and cell cycle pathways 8. Another gene set enriched in the CMS2 adenoma group was apical junction, this process also relates to increased proliferation. Additionally, CMS2 adenomas expressed genes involved in epithelial–mesenchymal transition, the transforming growth factor (TGF)‐β signalling pathway, and the development of muscles, which are processes typically assigned to CMS4 CRCs, and genes involved in the immune response (coagulation), which are characteristic of CMS1 CRCs. Considering that the enrichment analysis in the original CMS CRC characterisation was performed by comparing each CMS class with the other three CMS classes, the fact CMS1‐specific and CMS4‐specific processes arose in the CMS2 versus CMS3 comparison does not represent a contadictory result, as a different analysis was performed in this study. On the other hand, the majority of gene sets enriched in CMS3 adenomas were metabolism‐associated, including those involved in haem, fatty acid and sugar metabolism, which is in line with the original characterisation of the CMS3 ‘metabolic’ CRC subtype.

**Table 3:** Gene sets enriched in CMS2 and CMS3 adenomas

Gene set	Process category	Size	Members in signal	Normalised enrichment score	*P* value	FDR
Gene sets enriched in CMS2 adenomas in comparison with CMS3 adenomas						
G_2_M checkpoint	Proliferation	183	100	2.00	<0.001	<0.01
E2F targets	Proliferation	183	105	1.77	<0.001	0.01
MYC targets V2	Proliferation	57	78	1.57	0.005	0.02
Mitotic spindle	Proliferation	168	28	1.58	0.001	0.02
Epithelial–mesenchymal transition	Development	136	51	1.74	<0.001	0.01
Myogenesis	Development	103	42	1.67	0.001	0.01
PI3K–AKT–mTOR signalling	Signalling	85	38	1.62	0.004	0.01
Wnt–β‐catenin signalling	Signalling	33	13	1.61	0.005	0.02
TGF‐β signalling	Signalling	50	14	1.59	0.007	0.02
Coagulation	Immune	85	35	1.64	0.002	0.01
Apical junction	Cellular component	123	47	1.49	0.006	0.04
Gene sets enriched in CMS3 adenomas in comparison with CMS2 adenomas						
Protein secretion	Pathway	90	35	–1.78	<0.001	0.03
Glycolysis	Metabolic	169	45	–1.52	<0.001	0.08
Oxidative phosphorylation	Metabolic	194	85	–1.39	<0.001	0.13
Fatty acid metabolism	Metabolic	132	39	–1.35	0.017	0.13
Haem metabolism	Metabolic	144	33	–1.27	0.020	0.15
Oestrogen response late	Signalling	152	32	–1.30	0.020	0.15

Gene sets were grouped in process categories according to the original hallmark gene set grouping 32. Size indicates number of genes in the gene set; members in signal indicates how many genes from the gene set contributed to the enrichment score. The statistical values, normalised enrichment score, *P* values and FDR were calculated with GSEA 30. Gene sets enriched in CMS2 adenomas have positive enrichment scores, and gene sets enriched in CMS3 adenomas have negative enrichment scores.

To examine the differences between CMS2 and CMS3 adenomas in the context of CMS classes in CRC, ‘stromal scores’ and ‘immune scores’ from the ESTIMATE algorithm 33 and previously published ‘invasive front’ and ‘central tumour’ signature enrichments were calculated 35 (supplementary material, Figure S5). As expected, ‘stromal score’ and tumour ‘invasive front’ signatures showed a high level of enrichment in CMS4 CRCs as compared with adenomas and other CMS CRC classes. The ‘immune score’ was enriched in CMS1 cancers as compared with CMS2–3 lesions, whereas the ‘central tumour’ signature showed similar results for all groups.

### Validation in the independent series

Validation of the CMS classification results in colorectal adenomas was performed in an independent series – Series 3 (GSE20916) 29. Series 3 consists of colorectal adenomas (*n* = 45) and cancers (*n* = 36) measured on the Affymetrix array. To perform a similar analysis as that used for the study dataset, CRCs from the GSE39582 dataset (*n* = 566) were chosen as the reference dataset for batch effect removal, normalisation, and CMS classification 3. This reference dataset was the largest CRC series measured on the same platform as Series 3 and used in the original CMS classification publication 8. CMS classes were extracted for CRCs and adenomas from Series 3 (Table 2; supplementary material, Table S8). CMS classification of colorectal adenomas in Series 3 confirmed the results obtained with the study dataset, with most adenomas being labelled as CMS3 (*n* = 28, 62%), none as CMS4, and a small number as CMS1 (*n* = 1, 2%) or CMS2 (*n* = 2, 11%) (Table 2; supplementary material, Table S8). In Series 3, the distribution of the CMS classes among adenomas differed significantly from that of the cancers from the same series (*p* < 2.2 × 10^–16^). No significant differences between the distribution of CMS classes among adenomas from the study dataset and those from the validation set were observed (*p* = 0.13).

## Discussion

CMS classification constitutes an established consensus gene expression‐based subtyping of CRC. We set out to determine whether this molecular classification is already present at the adenoma stage. Classification of adenomas according to CMS was achieved for 54 of 62 adenomas, in a group‐wise analysis together with 59 CRCs from the study dataset and 556 TCGA CRC samples 15, 36. The results were validated in the independent series, in which 34 of 45 adenomas where classified with the same method; group‐wise analysis including 36 CRCs from the same series and 566 CRCs from the reference dataset [3,29].

The distribution of CMS classes in adenomas differed significantly from that in CRCs, in both the study dataset and the validation dataset. The vast majority (73% and 62% for the study and validation sets, respectively) of adenomas were classified as the ‘metabolic’ CMS3 type, which was the least frequent CMS class among CRCs from the study dataset (3%). Multiple gene expression profiling studies of colorectal adenomas and CRCs have shown upregulated metabolism in adenomas. In particular, pathway analysis of genes overexpressed in adenomas in comparison with cancers revealed the same pathways that were dysregulated in CMS3, including fatty acid, amino acid and sugar metabolism 8, 37, 38. It is evident that metabolic deregulation already occurs at the adenoma stage. In this study, GSEA comparing CMS2 and CMS3 adenomas confirmed enrichment of metabolic pathways in CMS3 adenomas. The results of this study imply that CMS3 is more representative of the adenoma than of the carcinoma stage. From the perspective of which adenomas have a risk of progressing to cancer, CMS3 may well represent low‐risk adenomas, which was confirmed by the enrichment of low‐risk adenomas in this class as defined by the presence of DNA copy number aberrations. As most adenomas never progress to cancer (95%), the observed frequency of CMS3 adenomas is consistent with this hypothesis.

Conversely, none of the adenomas from either the study dataset or the validation dataset were classified as the stroma‐rich poor‐prognosis CMS4 class. A process inherent to invasion and thus colorectal adenoma‐to‐carcinoma progression is activation of tumour stroma 21, 39. In fact, the tumour stroma represents an inflammatory response to foreign intruders, as well as being a scaffold for invading tumour cells. Mucosa of colorectal adenomas contains dysplastic epithelium as well as stroma (the lamina propria). In adenomas, this resembles the lamina propria of normal mucosa, being a framework of loose connective tissue, capillaries, myofibroblasts, and immune cells, and is quite different from the reactive stroma of cancers, which is the most prominent in CMS4 CRC. The lack of the mesenchymal subtype has also been observed for colorectal organoids, which are purely epithelial, and for patient‐derived xenografts, in which the stroma is of mouse origin 40, 41. Multiple studies have shown that the CMS4 signature is mostly driven by stroma rather than epithelial cancer cells 41, 42, 43. As the typical desmoplastic cancer stroma is, by definition, absent in adenomas, it is no surprise that no adenomas were classified as CMS4.

Regarding the CMS1 and CMS2 classes, the CMS classifier subtyped one of the adenomas with MSI as CMS1 and the second one as CMS3. MSI is rare in colorectal adenomas, with a prevalence of 3% overall 44, whereas approximately 15–20% of CRCs show MSI 45. The observations in the present study are consistent with these data. When colorectal adenomas acquire MSI, they are considered to progress rapidly, leaving a small window of opportunity for them to be detected, resulting in the low frequency of MSI in colorectal adenomas. Not all adenomas with MSI were classified as CMS1, consistent with the observations made on CRCs with MSI, a subset of which were also classified as CMS3 8. Specific features that discriminate CMS1 CRCs with MSI and CMS3 CRCs with MSI have not been described yet. In the validation set, one adenoma was classified as CMS1 as well, but the MSI status of this adenoma is unknown. Eight of the adenomas were classified as CMS2 in the study dataset, and five in the validation set. From the perspective of adenoma‐to‐carcinoma progression, this is particularly interesting, as CMS2 represents canonical CRC carcinogenesis. Given that Wnt and MYC pathway activation occurs mostly in the transition from normal epithelium to adenoma, it may seem unexpected that CMS2 is not the predominant class within adenomas 46. On the assumption that not the sequential order but the accumulation of mutations causes tumour progression, there must be more alterations in these adenomas to be classified as CMS2. Indeed, the enrichment of high‐risk adenomas within CMS2 suggests that CMS2 adenomas might be closer to becoming malignant than those classified as CMS3. Additionally, the chromosomal gain of 20q and loss of 18q were found to occur more often in CMS2 adenomas. Gain of 20q is associated with a gene dosage effect of multiple genes 16, including *AURKA* and *TPX2*, which play a role in the G_2_/M phase of the cell cycle 47. This is consistent with the observed enrichment of the G_2_/M checkpoint and mitotic spindle assembly gene sets in CMS2 adenomas. Another characteristic specific for adenoma‐to‐carcinoma progression and the CMS2 adenoma class is upregulation of pathways such as the cell cycle and epithelial differentiation 21. In this study, GSEA confirmed that CMS2 adenomas have increased expression of genes involved in proliferation, the cell cycle and even epithelial–mesenchymal transition as compared with CMS3 adenomas. These findings are in line with CMS2 CRC characterisation as well as with the biological processes required for adenoma‐to‐carcinoma progression. Our results suggest that CMS2 adenomas, rather than CMS3 adenomas, may represent lesions at risk of becoming malignant. Owing to the lack of copy number information in Series 3, the association between risk of progression and CMS classification could not be further validated. Nevertheless, this association should be further investigated. Adenomas, once detected during colonoscopy, are completely removed, thereby interrupting their natural history in terms of either progressing to cancer or not. Currently, adenoma‐to‐carcinoma progression can only be studied *in vitro* by the use of, for example, organoid models. Although this has been done by perturbing frequently mutated cancer genes with prominent roles in CRC pathogenesis 48, relevant aspects of adenoma‐to‐carcinoma progression, including CIN, still remain to be incorporated in these model system studies.

The CMS classification of cancers revealed a relatively large number of CMS4 cases in the present series. Taking into account the different sample sizes of the current study and the original CMS publication, and given the variation in distributions of CMS classes among the six datasets from which the CMS classification originated 1, 2, 3, 5, 6, 7, 8, it may be that the CMS class distribution varies per dataset.

In the study dataset, we used large adenomas to sample fresh frozen material for research purposes, as well as routine tissue processing for diagnostics. Therefore, the majority (95%) of the adenomas were > 1 cm. Given the association of adenoma size with progression risk 49, the proportion of CMS3 could be even higher in smaller adenomas. The current study, however, does not allow conclusions to be drawn about the stage of development from normal epithelium to adenoma at which a CMS signature becomes detectable.

The present study focused on conventional adenomas, which are the most common precursors of CRC, especially in the context of CIN 50, representing the classic adenoma‐to‐carcinoma progression model. More recently, a serrated pathway has been introduced, with sessile serrated lesions being precursors of CRC 50. The CMS classification of these lesions has already been presented besides the CMS classification of a small number of tubular adenomas, and resulted in a different distribution of the CMS classes from that observed in the current study 51. However, given the highly selective composition of adenomas in this dataset and its considerable differences from our study cohort, significant variation in CMS classification is to be expected.

Technically, a combined analysis of the study dataset and the TCGA CRC series was performed to reduce the effect of the RNA‐seq data normalisation on the CMS classification. Additionally, because of a further normalisation step implemented in the random forest CMS algorithm, combined analysis reduced the impact of the potentially different distribution of CMS classes in the study dataset from that in the original CMS training set. The concept of batch effect adjustment to a ‘gold‐standard’ dataset, which the model was trained on, and classification by use of a merged dataset was previously introduced 26. This approach proved to be appropriate for our research question by providing stability to the classifier in comparison with applying it on the study dataset alone (data not shown). The CMS classification of TCGA data performed in this study was not biased by our approach, as the original CMS labels for these samples were reassigned with an accuracy of 97%. Additionally, the CMS classification results for the adenomas were largely reproduced with the SSP CMS classifier (supplementary material, Tables S9 and S10). The SSP method is not sensitive to the composition of the dataset on which it is applied, so it did not require the context of a large series of CRCs or batch effect removal. Therefore, it is suitable for validation of the entire data analysis approach. The SSP method confirmed the CMS classes of adenomas to a large extent; however, in some cases, it lacked confidence in recognising CMS1 or CMS2 expression traits.

So far, classification of colorectal neoplasia has been morphology‐based. Adenomas are classified on the basis of histological type, size and grade of dysplasia, whereas cancers are subtyped on the basis of grade of differentiation and stage. The CMS classification is an approach for molecular classification of cancers based on RNA expression. The present study has extended this approach to colorectal adenomas, and has demonstrated that CMS classification can be effectively applied to these lesions. In conclusion, colorectal adenomas proved to be heterogeneous in terms of CMS class, but with a different distribution from that of cancers. CMS3 turned out to be the most prevalent among the conventional adenomas, and our results indicate that it may represent mostly adenomas at low risk of progressing to CRC as compared with CMS1 or CMS2 adenomas. The frequency of CMS classes observed in adenomas is consistent with what could be expected on the basis of differences between adenomas and carcinomas, and on the proportion of adenomas expected to progress to cancer.

## Author contributions statement

MAK, LJWB, GB, YH, APS, GJ, BC, LFAW, CR, RJAF and GAM conceived the study and the experiments. NCTvG and GAM performed the histopathological review. LJWB, ASB, PDvD and MdJ performed experiments. MAK, GB and LFAW contributed to the design of the data analysis. MAK and CR performed the bioinformatics analysis. All authors were involved in writing the paper and gave final approval to the submitted and published versions.

**SUPPLEMENTARY MATERIAL ONLINE**

**Supplementary materials and methods**

**Figure S1.** Multidimensional scaling of the Euclidian distance between the gene expression profiles of all the samples from the study dataset with TCGA

**Figure S2.** Hierarchical clustering based on the gene expression profiles of the top 1000 most variable genes

**Figure S3.** Multidimensional scaling of the Euclidian distance between the gene expression profiles of all the samples for the validation set

**Figure S4.** Hierarchical clustering based on the gene expression profiles of the top 1000 most variable genes for the validation set

**Figure S5.** ESTIMATE scores and ssGSEA enrichment scores among CMS classes in adenomas and cancer

**Table S1.** Availability of the study data

**Table S2.** Comparison of the CMS classification of the TCGA data set in the current study to the original TCGA CMS labels

**Table S3.** CMS classification of colorectal cancers from the study dataset

**Table S4.** MSI samples in the study dataset: association between CMS classes in CRCs and differentiation grade, stage and MSI status

**Table S5.** CMS classification of adenomas from the study dataset

**Table S6.** Cancer‐associated events (CAEs): DNA copy number aberrations and the risk of progression for adenomas in the study dataset

**Table S7.** Fisher exact test results for the association analysis in adenomas from the study dataset

**Table S8.** CMS classification of adenomas and cancers from the validation set

**Table S9.** CMS classification of colorectal adenomas from the study dataset performed with Single Sample Predictor

**Table S10.** Comparison of the CMS classification of colorectal adenomas by the study approach (random forest CMS classifier) and single sample predictor

## Supporting information


**Appendix S1**. Supplementary Materials and Methods

**Figure S1. Multidimensional scaling of the Euclidian distance between the gene expression profiles of all the samples from the study dataset with TCGA.** (A) Plot before batch effect removal. Three separate batches can be clearly distinguished, with white dots representing samples from the TCGA dataset, blue from Series 1 and red from Series 2. (B) Plot after batch effect removal. Samples originating from different datasets cannot be distinguished by their locations on the plot, indicating that the batch effect was removed.
**Figure S2. Hierarchical clustering based on the gene expression profiles of the top 1000 most variable genes.** (A) Heatmap of all three datasets before batch effect removal. The batches corresponding to the TCGA dataset, Series 1 and Series 2 can be distinguished in the heatmap. (B) Heatmap before batch correction of the Series 1 and Series 2 study datasets only. Next to the two batches, one can distinguish clusters enriched with adenomas and clusters enriched with cancers. (C) Heatmap of all three datasets after batch effect removal. Samples from the three experiments do not cluster together. (D) Heatmap of the Series 1 and Series 2 study datasets after batch effect removal. Clusters enriched with adenomas or cancers can still be distinguished, meaning that batch effect correction did not remove the variability between different lesions.
**Figure S3. Multidimensional scaling of the Euclidian distance between the gene expression profiles of all the samples for the validation set.** Series 3 is the validation set with colorectal adenomas and cancers. Reference is the reference series with only colorectal cancers. (A) Plot before batch effect removal. Two separate batches can be clearly distinguished, with white dots representing samples from the reference dataset and blue dots from the Series 3. (B) Plot after batch effect removal. The samples originating from different datasets cannot be distinguished by their location on the plot, indicating that the batch effect was removed.
**Figure S4.** Hierarchical clustering based on the gene expression profiles of the top 1000 most variable genes. Reference is the reference dataset used for normalisation, Series 3 is the validation set. (A) Heatmap of the two datasets before batch effect removal. The batches corresponding to the Reference and Series 3 can be distinguished in the heatmap. (B) Heatmap before batch correction of the Series 3 only. Clusters enriched with adenomas and clusters enriched with cancers can be distinguished. (C) Heatmap of the two datasets after batch effect removal. Samples from the two experiments do not cluster together. (D) Heatmap of the Series 3 after batch effect removal. Clusters enriched with adenomas or cancers can still be distinguished, meaning that batch effect correction did not remove the variability between different lesions.
**Figure S5.** ESTIMATE scores and ssGSEA enrichment scores among CMS classes in adenomas and cancer. “Stromal” (A) and “Immune” (B) scores were calculated using the ESTIMATE algorithm and plotted per CMS group in colorectal adenomas and cancers. “Invasive Front” (C) and “Central Tumor” (D) enrichment was calculated using the ssGSEA algorithm.

**Table S1.** Availability of the study data
**Table S2.** Comparison of the CMS classification of the TCGA data set in the current study to the original TCGA CMS labels
**Table S3.** CMS classification of colorectal cancers from the study dataset
**Table S4.** MSI samples in the study dataset: association between CMS classes in CRCs and differentiation grade, stage and MSI status
**Table S5.** CMS classification of adenomas from the study dataset
**Table S6.** Cancer‐associated events (CAEs): DNA copy number aberrations and the risk of progression for adenomas in the study dataset
**Table S7.** Fisher exact test results for the association analysis in adenomas from the study dataset
**Table S8.** CMS classification of adenomas and cancers from the validation set
**Table S9.** CMS classification of colorectal adenomas from the study dataset performed with Single Sample Predictor
**Table S10.** Comparison of the CMS classification of colorectal adenomas by the study approach (random forest CMS classifier) and single sample predictor
