# Supplementary material for: Consensus molecular subtype classification of colorectal adenomas
Source: J Pathol. 2018 Aug 31;246(3):266–76. doi: 10.1002/path.5129 (PMC6221003; doi:10.1002/path.5129)
Supplement: Supplementary file 1 — Appendix S1. Supplementary Materials and Methods [file PATH-246-266-s001.docx]

## Consensus molecular subtypes classification of colorectal adenomas

## Supplementary Materials and Methods

## Sample collection

Series 1 originated from the NGS-ProToCol dataset [36]; 60 snap-frozen colorectal tumours (30 colorectal polypoid adenomas and 30 colorectal carcinomas) were collected at the department of Pathology of the VU University Medical Centre in Amsterdam, between 2011 and 2014. Excluded were patients below the age of 50, patients with Lynch syndrome, or patients known to have received radio- or chemotherapy before tumour removal. Samples were reviewed by an expert gastrointestinal pathologist and classified according to standard histopathological criteria. DNA and RNA were isolated from snap-frozen tissue pieces (Supplementary Materials and Methods). For Series 2, 32 colorectal polypoid adenomas and 29 colorectal carcinomas were collected at the department of Pathology of the VU University Medical Centre in Amsterdam and described in a previous study [22]. RNA isolated from fresh frozen specimens of these samples was available.

## DNA and RNA isolation from fresh frozen tissue

Series 1: DNA and RNA were isolated from snap-frozen tissue pieces. For each piece, a “before-” and “after-isolation” H&E slide was made. In between, tissue slices of 25 μm (for RNA isolation) or 15 μm (for DNA isolation) were cut. The H&E slides were reviewed by an expert gastrointestinal pathologist. For most of the tissues (*n* = 54) at least 70% of the tissue contained tumour cells. For six of them the tumour cell percentage was 60%. RNA was isolated from 30–40 25-μm slides using the miRNeasy Mini kit (QIAgen, Cat no 217004). The cut tissues were homogenized in 700 μl TRIzol (Invitrogen, cat. no. 15596026), vortexed and incubated for 15–20 minutes at room temperature, following the manufacturer’s protocol. Genomic DNA was isolated from 15–25 15-μm slides, using the AllPrep DNA/RNA/miRNA Universal Kit (QIAgen, 80224), following the manufacturer's protocol. DNA and RNA concentrations and purities were measured on a Nanodrop ND-1000 spectrophotometer (ThermoFisher Scientific). For the DNA samples, the double stranded DNA (dsDNA) concentrations were also measured using the Qubit 3.0 Fluorometer using the dsDNA HS Assay Kit (Invitrogen, Cat no Q32851) DNA and RNA samples were also analyzed on a 0.8–1% agarose gel to confirm high molecular weight DNA or to check the RNA integrity.

## Low-coverage whole genome sequencing and DNA copy number analysis

DNA from Series 1 was subjected to low-pass whole genome sequencing. The amount of dsDNA in the genomic DNA samples was quantified by using the Qubit, dsDNA HS Assay Kit (Invitrogen, Cat no Q32851). Up to 500 ng of dsDNA were fragmented by Covaris shearing to obtain fragment sizes of 160–180 bp. Samples were purified using 1.6X Agencourt AMPure XP PCR Purification beads according to manufacturer’s instructions (Beckman Coulter, Cat no A63881). The sheared DNA samples were quantified and qualified on a BioAnalyzer system using the DNA7500 assay kit (Agilent Technologies, cat no. 5067- 1506). With an input of maximum 1 μg sheared DNA, library preparation for Illumina sequencing was performed using the KAPA HTP Library Preparation Kit (KAPA Biosystems, Cat no KK8234). During library enrichment four to six PCR cycles were used to obtain enough yield for sequencing. After library preparation, the libraries were cleaned up using 1X AMPure XP beads. All DNA libraries were analyzed on a Caliper LabChip GX system with the HT DNA HiSense Reagent Kit (Caliper Life Sciences Inc, cat no. CLS760672) for determining the molarity. Up to 78 uniquely indexed samples were mixed together by equimolar pooling, in a final concentration of 10 nM, and subjected to sequencing on an Illlumina HiSeq 2500 machine in three lanes of a single read 65-bp run using v.4 chemistry, according to manufacturer’s instructions. On average over 10 million reads were obtained per adenoma sample.

Low quality reads and adapter sequences were trimmed with Trimmomatic version 3 to an average quality score for sliding window of 24 and quality of 26 both at the beginning and at the end of the sequences [52]. Reads were cropped to a length of 50 bp; shorter reads were removed. Trimmed reads were uniquely aligned to the human reference genome build hg19 using Burrows-Wheeler Aligner (“bwa aln”, allowing two mismatches and end-trimming of bases with qualities below 40, and “bwa samse” with default parameters) [53]. Reads identifiable as PCR duplicates were filtered out using Picard Tools MarkDuplicates version 2.7.1 [54]. Read counting per bins, normalizations, corrections and filtering were done with Bioconductor package QDNAseq [55]. After median normalization, wave-correction was performed with an R package NoWaves [56]. Copy number segmentation was performed using Bioconductor package DNAcopy [57]. Gained and lost regions were identified using Bioconductor package CGHcall [58]. Copy number aberrations called with probability of more than 0.5 were taken along in further analysis.

## RNA sequencing and data pre-processing

Series 1: The NEBNext Ultra Directional RNA Library Prep Kit for Illumina with rRNA reduction was used to process the samples. Sample preparation was performed according to the protocol "NEBNext Ultra Directional RNA Library Prep Kit for Illumina" (NEB #E7420S/L and NEB #E6310S/L/X). Briefly, rRNA was reduced using a ribonuclease H-based method. Then, fragmentation of the rRNA-reduced RNA and a cDNA synthesis was performed. This was used for ligation with the sequencing adapters and PCR amplification of the resulting product. The quality and yield after sample preparation was measured with the Fragment Analyzer (Advanced Analytical). Clustering and DNA sequencing using the Illumina cBot and HiSeq 2500 were performed according manufacturer’s protocols. A concentration of 16.0 pM of DNA was used as input. HiSeq control software HCS v2.2.58 was used. Image analysis, base calling, and quality check were performed with the Illumina data analysis pipeline RTA v1.18.64 and Bcl2fastq v2.17. On average 67 million reads were obtained per sample.

Series 2: Quality and quantity of the total RNA were assessed by the 2100 Bioanalyzer using a Nano chip (Agilent). Total RNA samples having a RIN > 8 were subjected to library generation. Strand-specific libraries were generated using the TruSeq Stranded mRNA sample preparation kit (Illumina Inc., Cat no RS-122-2101/2) according to the manufacturer's instructions (Illumina Inc., Cat no 15031047 Rev. E). Briefly, polyadenylated RNA from intact total RNA was purified using oligo(dT) beads. Following purification, the RNA was fragmented, random-primed and reverse transcribed using SuperScript II Reverse Transcriptase (Invitrogen, Cat no 18064-014) with the addition of actinomycin D. Second strand synthesis was performed using polymerase I and ribonuclease H with replacement of dTTP for dUTP. The generated cDNA fragments were 3′-end adenylated and ligated to Illumina paired-end sequencing adapters and subsequently amplified by 12 cycles of PCR. The libraries were analyzed on a 2100 Bioanalyzer using a 7500 chip (Agilent), diluted and pooled equimolar into a 10 nM multiplex sequencing pool, containing 18 samples per pool. RNA sequencing was performed on an Illumina HiSeq V4 2500, using a 125 bases paired-end run. On average 32 million reads were obtained per sample.

RNA-seq data preprocessing was performed for each series as follows. Low quality reads and adapter sequences were trimmed by Trimmomatic [52] version 3 to average quality score for sliding window of 24, and 26 for both leading and trailing part of the sequences. Minimum length was set to 36 bases. Mapping was performed with the STAR aligner [59] version 2.4.2a to the human genome (USCS RefSeq hg38, annotation gencode v22 [60]). Read counts per transcript were obtained with featureCounts from the Subread package v1.5.0-p2 [61] with the gencode v22 annotation as reference. RPKM values were obtained with the use of the rpkm function from the edgeR Bioconductor package version 3.12.1 [62] and log_2_ transformed.

## The Cancer Genome Atlas CRC data

Gene expression data in the form of FPKM values were downloaded on 26.01.2017 from the NCI Genomic Data Commons (GDC) portal Release 1.4.1 for all the TCGA colorectal cancer samples (COAD and READ projects) [15,63]. The dataset was filtered for only primary tumours. 557 TCGA sample labels used by the CRC Subtyping Consortium in the original CMS classification were obtained from their Synapse instance [8,28] and used to filter the TCGA dataset; one TCGA label was missing from the GDC data portal. For the 556 TCGA samples FPKM values were log_2_ transformed, forming a reference dataset for data normalization and CMS classification.

## Validation set

CEL files were downloaded for datasets GSE20916 and GSE39582 from Gene Expression Omnibus and reprocessed to reflect current knowledge on the Ensembl genome annotation. Probe sequences were realigned to the latest Ensembl genome version using the brainarray framework (first introduced by Dai et al. [64]), and re-normalization was performed using custom rma (affy). Ensembl IDs were translated to gene symbols with the use of the latest edition of biomaRt version (ensembl 88, March 2017). The batch effect was removed with the use of M-Combat [26] as performed for Series 1 and 2 with the following changes; GSE39582 served as the reference dataset while Series 3 was the normalized batch. Evaluation of the batch effect removal and preservation of the differences between the adenomas and the cancers was performed with the multidimensional scaling algorithm on the Euclidian distance between the expression profiles and hierarchical clustering with complete linkage on the log_2_ expression values of the top 1000 variable genes (supplementary material, Figures S3, S4), respectively. Gene symbols were translated into Entrez ID with the use of the biomaRt Bioconductor package [27]. Reference dataset and Series 3 were merged after batch effect removal and CMS classification with the random forest algorithm was performed on the merged dataset. CMS classes were assigned as for the study dataset.
