# Supplementary material for: Consensus molecular subtype classification of colorectal adenomas
Source: J Pathol. 2018 Aug 31;246(3):266–76. doi: 10.1002/path.5129 (PMC6221003; doi:10.1002/path.5129)
Supplement: Supplementary file 2 — Figure S1. Multidimensional scaling of the Euclidian distance between the gene expression profiles of all the samples from the study dataset with TCGA. (A) Plot before batch effect removal. Three separate batches can be clearly distinguished, with white dots representing samples from the TCGA dataset, blue from Series 1 and red from Series 2. (B) Plot after batch effect removal. Samples originating from different datasets cannot be distinguished by their locations on the plot, indicating that the batch effect was removed. Figure S2. Hierarchical clustering based on the gene expression profiles of the top 1000 most variable genes. (A) Heatmap of all three datasets before batch effect removal. The batches corresponding to the TCGA dataset, Series 1 and Series 2 can be distinguished in the heatmap. (B) Heatmap before batch correction of the Series 1 and Series 2 study datasets only. Next to the two batches, one can distinguish clusters enriched with adenomas and clusters enriched with cancers. (C) Heatmap of all three datasets after batch effect removal. Samples from the three experiments do not cluster together. (D) Heatmap of the Series 1 and Series 2 study datasets after batch effect removal. Clusters enriched with adenomas or cancers can still be distinguished, meaning that batch effect correction did not remove the variability between different lesions. Figure S3. Multidimensional scaling of the Euclidian distance between the gene expression profiles of all the samples for the validation set. Series 3 is the validation set with colorectal adenomas and cancers. Reference is the reference series with only colorectal cancers. (A) Plot before batch effect removal. Two separate batches can be clearly distinguished, with white dots representing samples from the reference dataset and blue dots from the Series 3. (B) Plot after batch effect removal. The samples originating from different datasets cannot be distinguished by their location on the plot, indicating that the b [file PATH-246-266-s002.pdf]

Figure S1

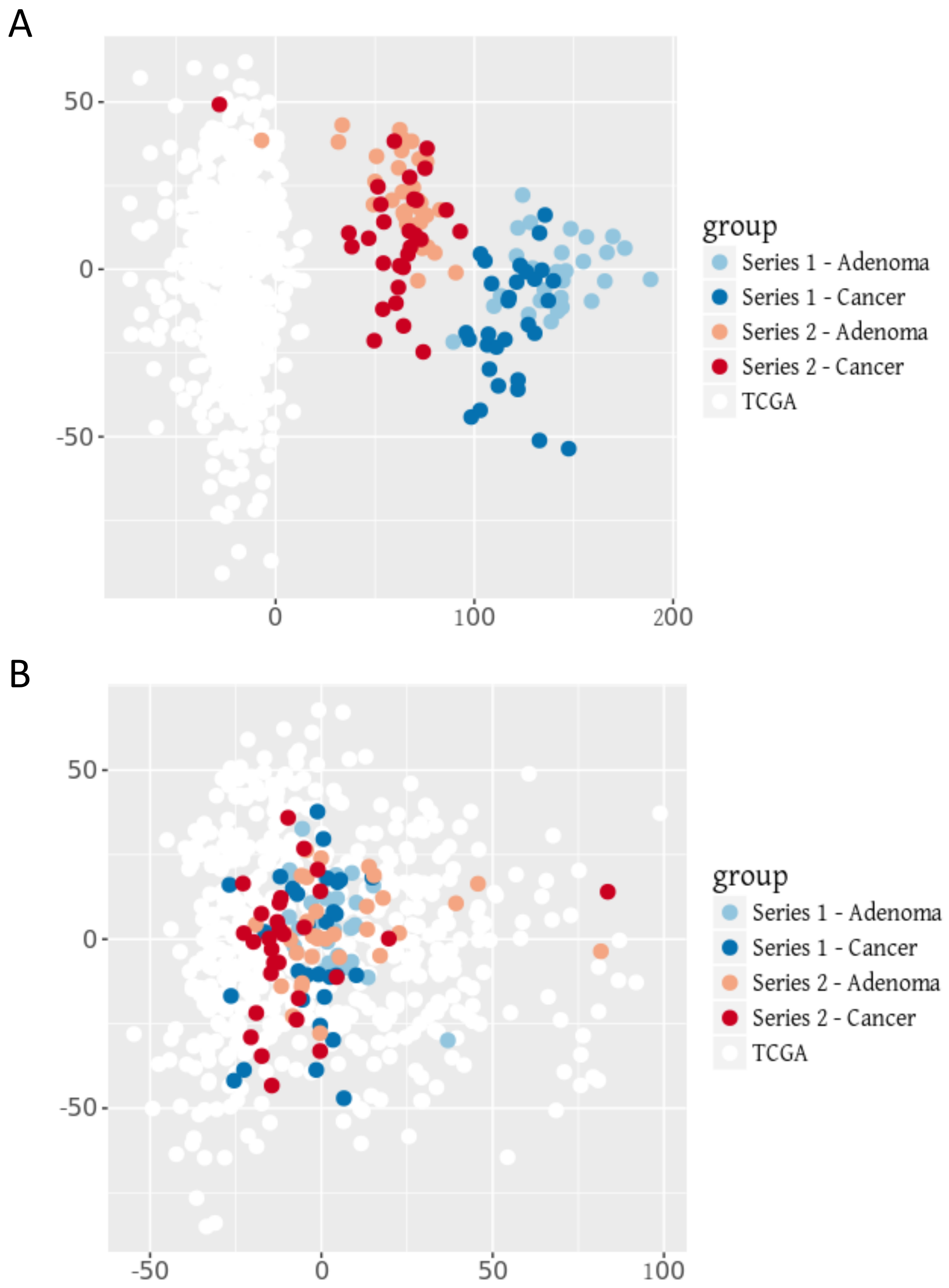

Figure S1. Multidimensional scaling of the Euclidian distance between the gene expression profiles of all the samples from the study dataset with TCGA. (A) Plot before batch effect removal. Three separate batches can be clearly distinguished, with white dots representing samples from the TCGA dataset, blue from Series 1 and red from Series 2. (B) Plot after batch effect removal. Samples originating from different datasets cannot be distinguished by their locations on the plot, indicating that the batch effect was removed.

Figure S2

A

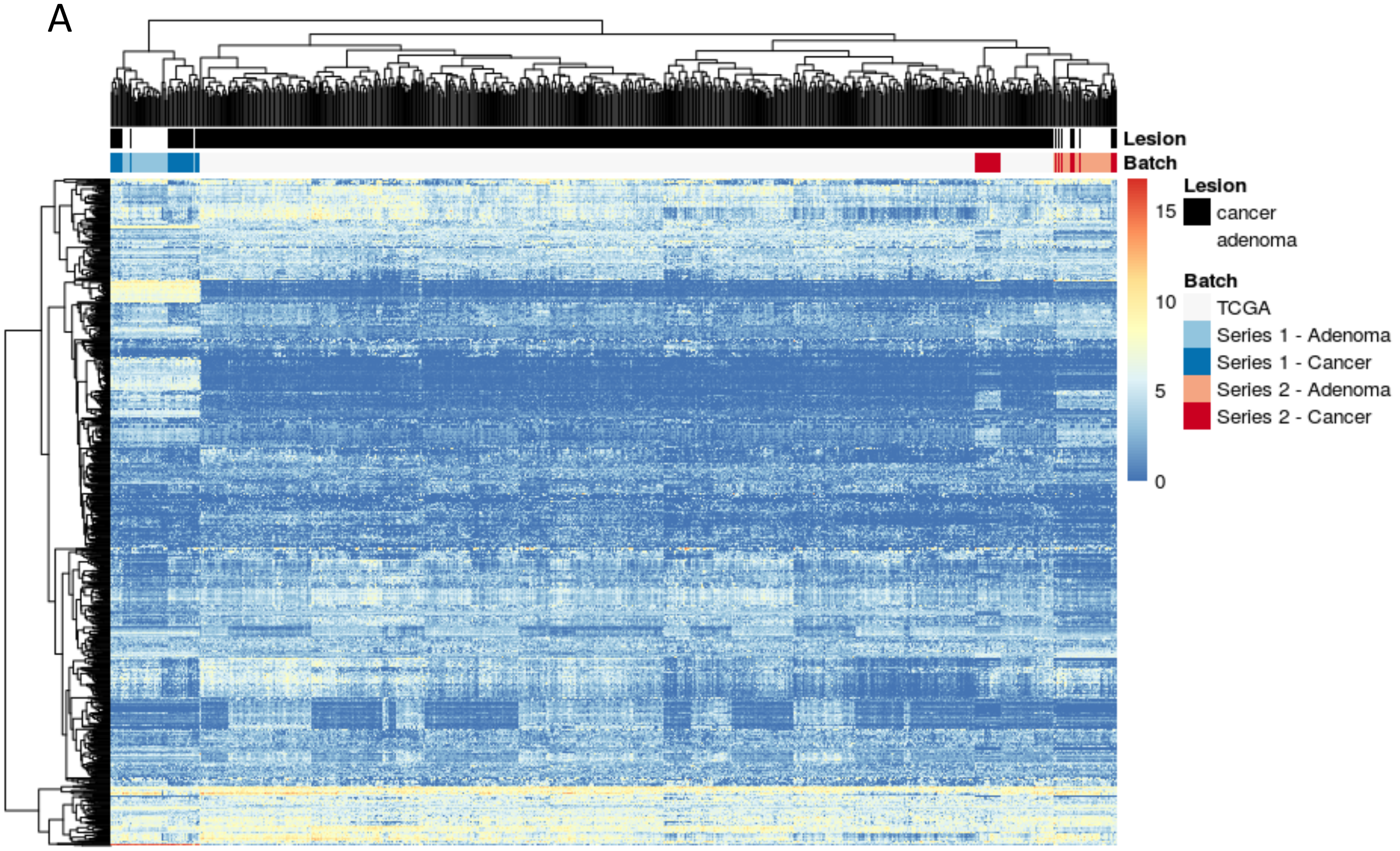
Lesion
Batch
15
10
5
0
Lesion
cancer
adenoma
Batch
TCGA
Series 1 - Adenoma
Series 1 - Cancer
Series 2 - Adenoma
Series 2 - Cancer

B

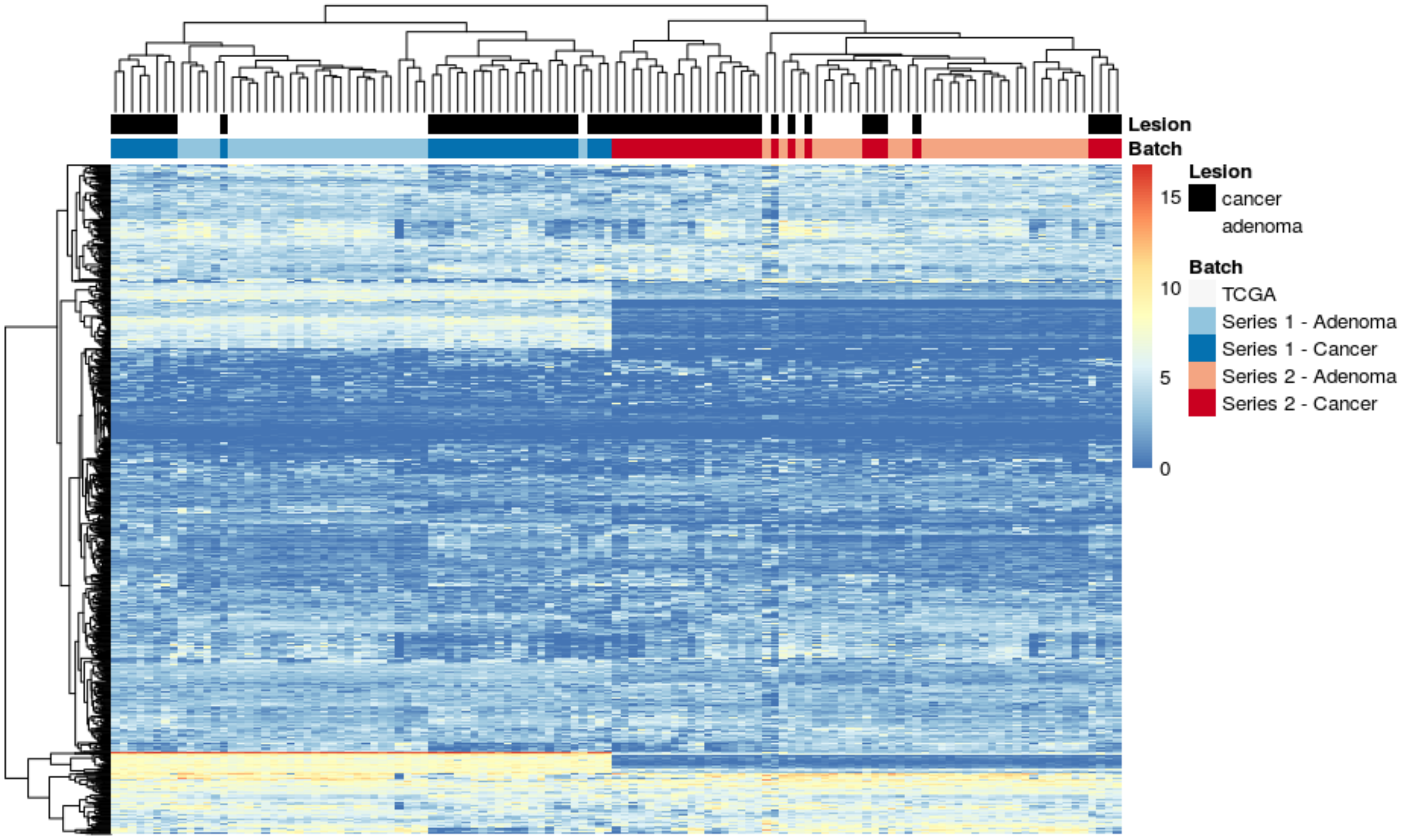
Lesion
Batch
15
10
5
0
Lesion
cancer
adenoma
Batch
TCGA
Series 1 - Adenoma
Series 1 - Cancer
Series 2 - Adenoma
Series 2 - Cancer

Figure S2

C

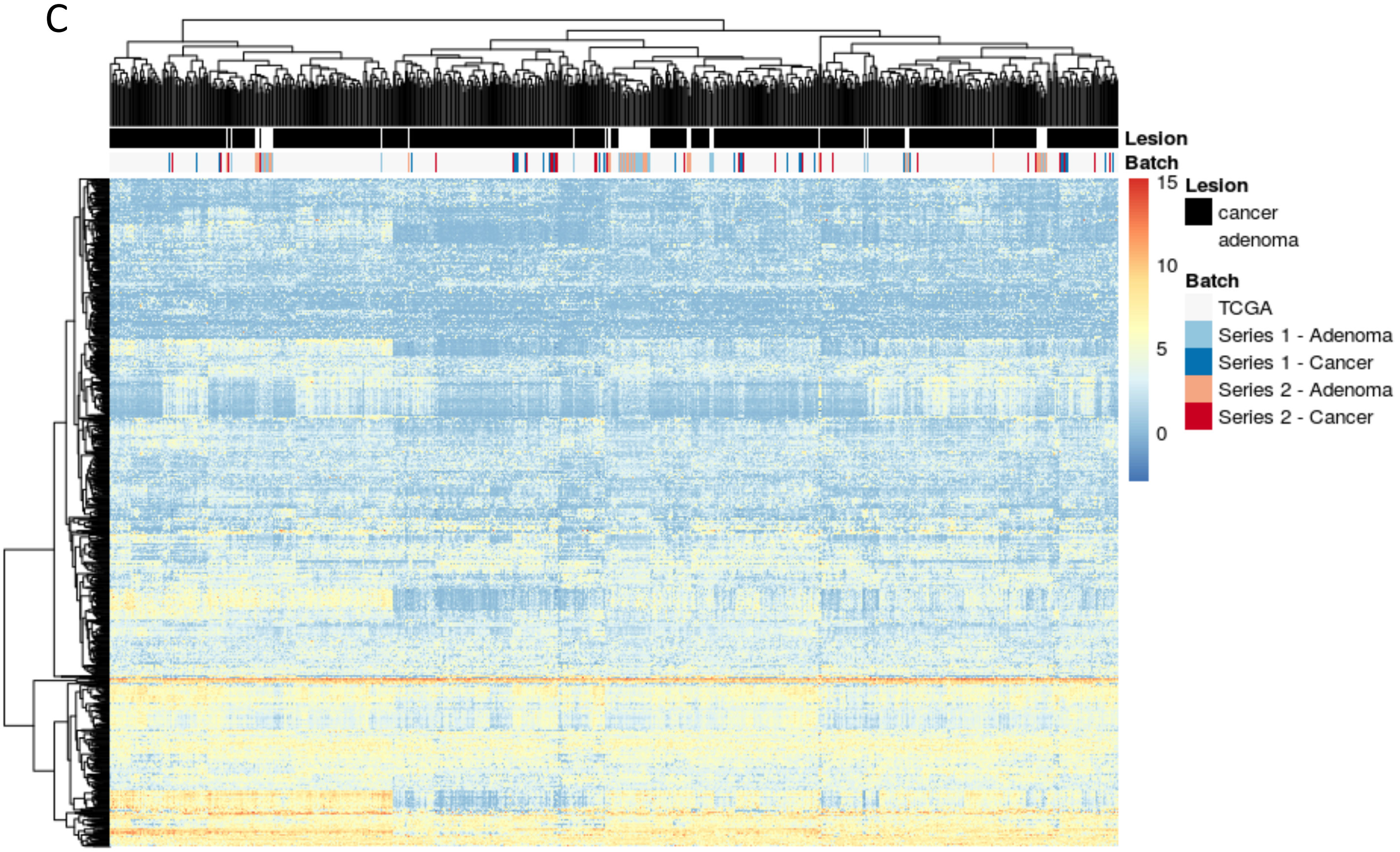
Lesion
Batch
15
10
5
0
Lesion
cancer
adenoma
Batch
TCGA
Series 1 - Adenoma
Series 1 - Cancer
Series 2 - Adenoma
Series 2 - Cancer

D

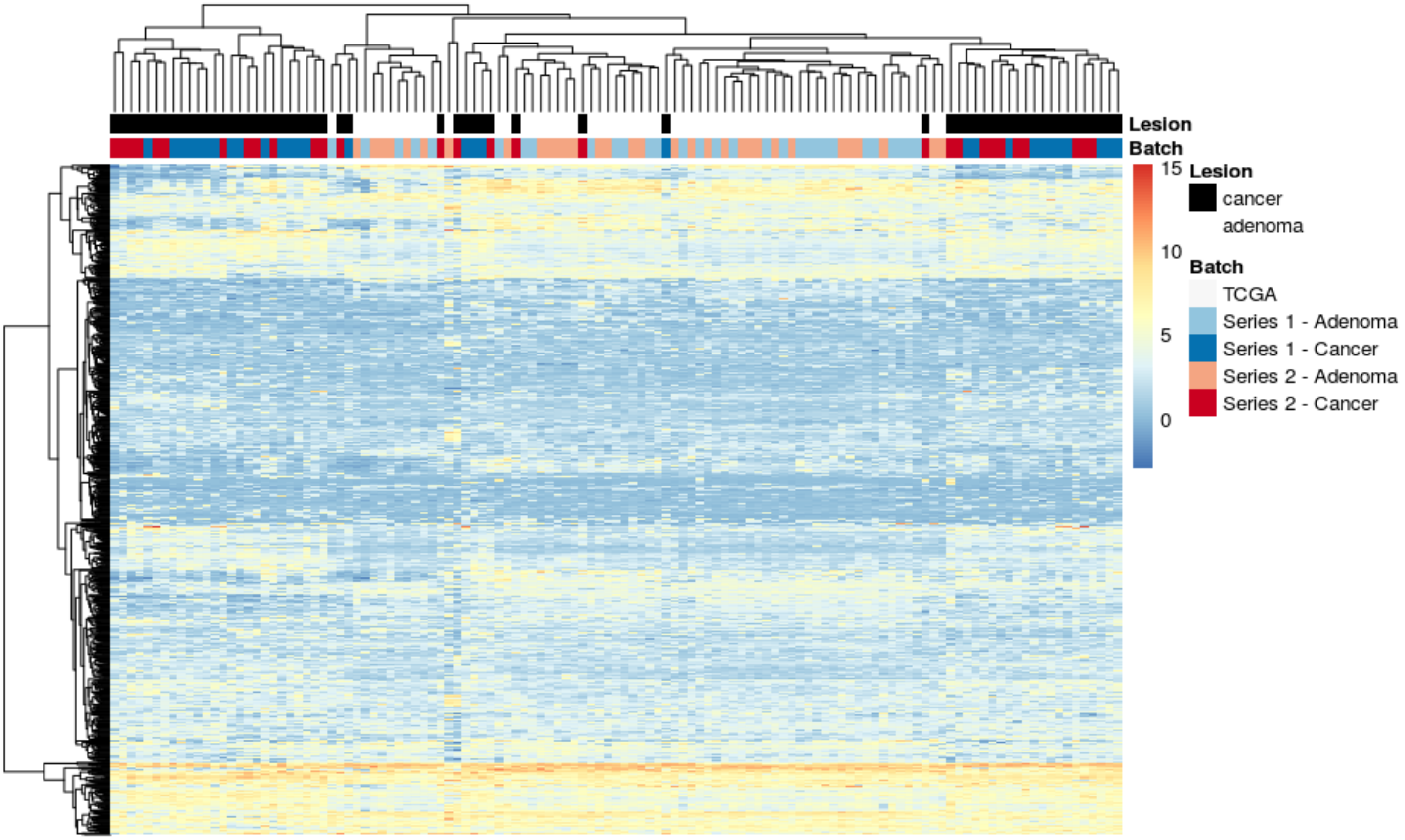
Lesion
Batch
15
10
5
0
Lesion
cancer
adenoma
Batch
TCGA
Series 1 - Adenoma
Series 1 - Cancer
Series 2 - Adenoma
Series 2 - Cancer

Figure S2. Hierarchical clustering based on the gene expression profiles of the top 1000 most variable genes. (A) Heatmap of all three datasets before batch effect removal. The batches corresponding to the TCGA dataset, Series 1 and Series 2 can be distinguished in the heatmap. (B) Heatmap before batch correction of the Series 1 and Series 2 study datasets only. Next to the two batches, one can distinguish clusters enriched with adenomas and clusters enriched with cancers. (C) Heatmap of all three datasets after batch effect removal. Samples from the three experiments do not cluster together. (D) Heatmap of the Series 1 and Series 2 study datasets after batch effect removal. Clusters enriched with adenomas or cancers can still be distinguished, meaning that batch effect correction did not remove the variability between different lesions.

Figure S3

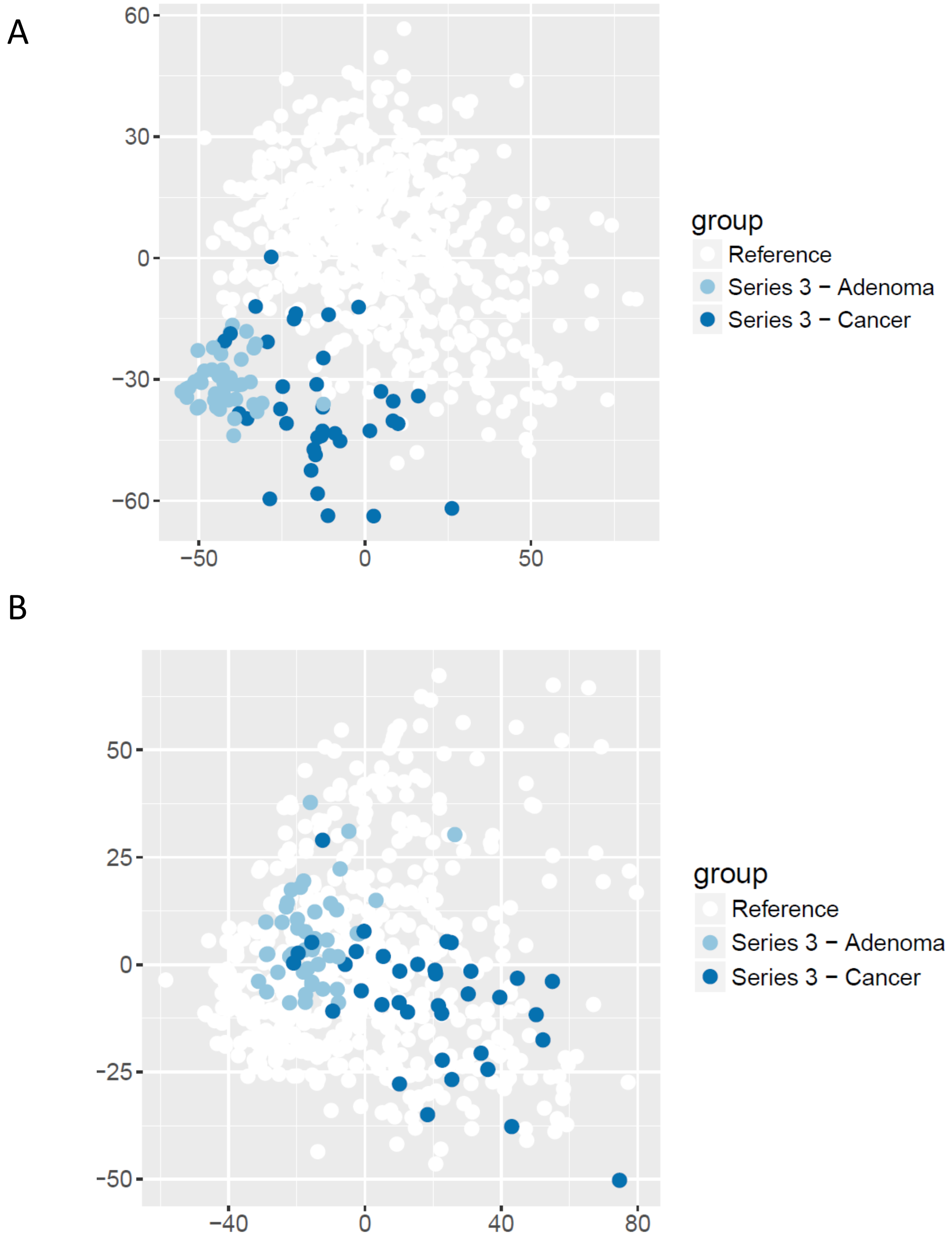

Figure S3. Multidimensional scaling of the Euclidian distance between the gene expression profiles of all the samples for the validation set. Series 3 is the validation set with colorectal adenomas and cancers. Reference is the reference series with only colorectal cancers. (A) Plot before batch effect removal. Two separate batches can be clearly distinguished, with white dots representing samples from the reference dataset and blue dots from the Series 3. (B) Plot after batch effect removal. The samples originating from different datasets cannot be distinguished by their location on the plot, indicating that the batch effect was removed.

Figure S4

A

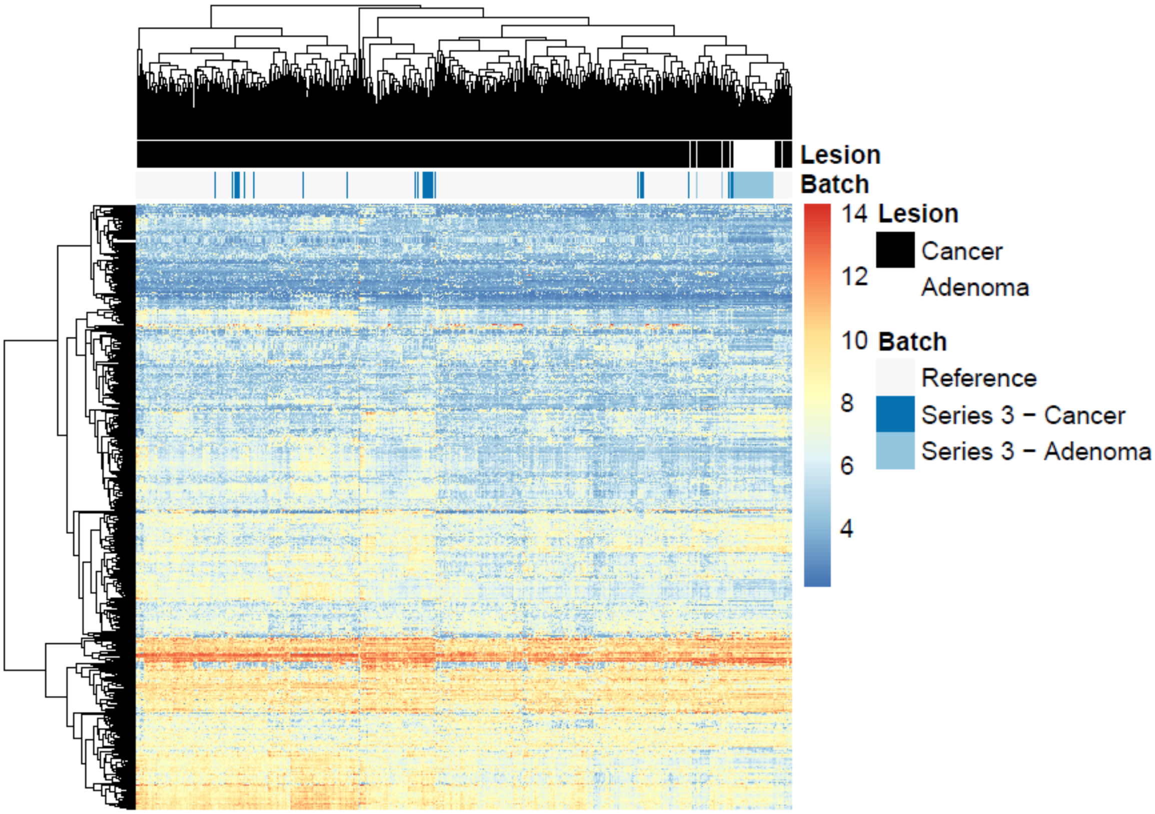
Lesion
Batch
14
12
10
8
6
4
Lesion
Cancer
Adenoma
Batch
Reference
Series 3 − Cancer
Series 3 − Adenoma

B

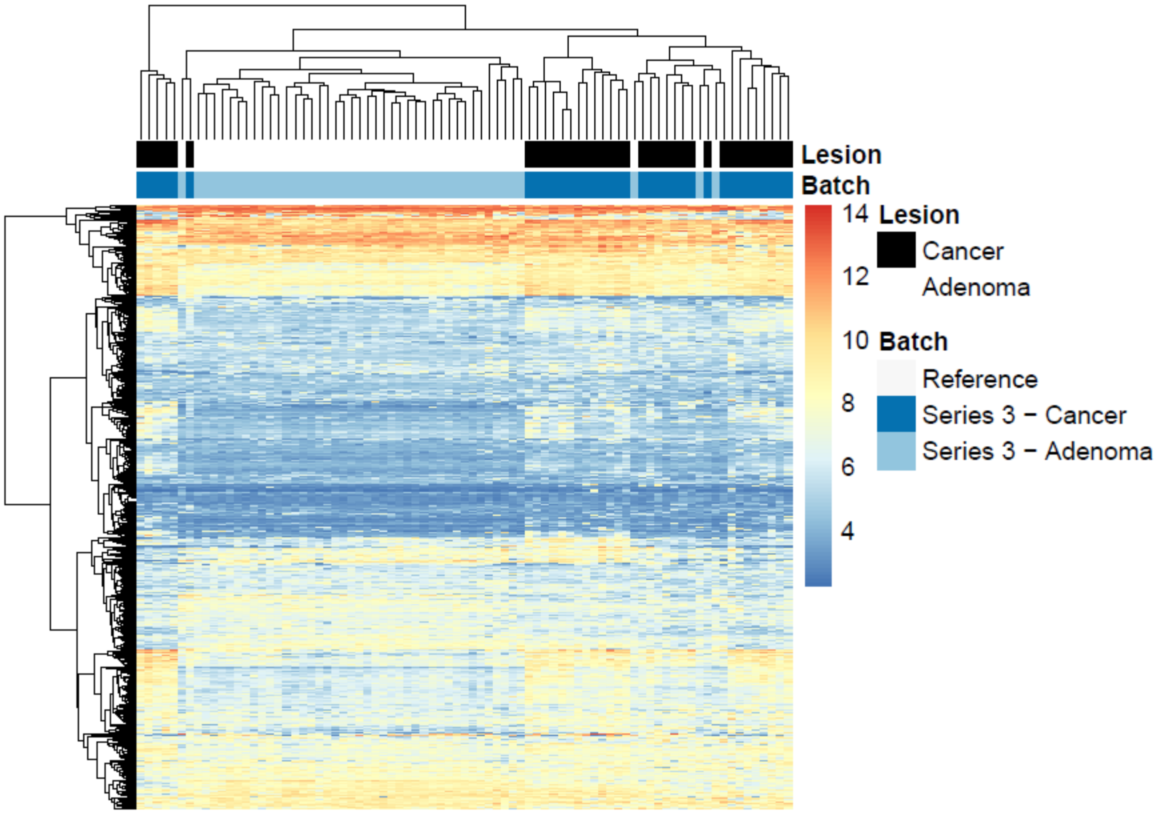
Lesion
Batch
14
12
10
8
6
4
Lesion
Cancer
Adenoma
Batch
Reference
Series 3 − Cancer
Series 3 − Adenoma

Figure S4

C

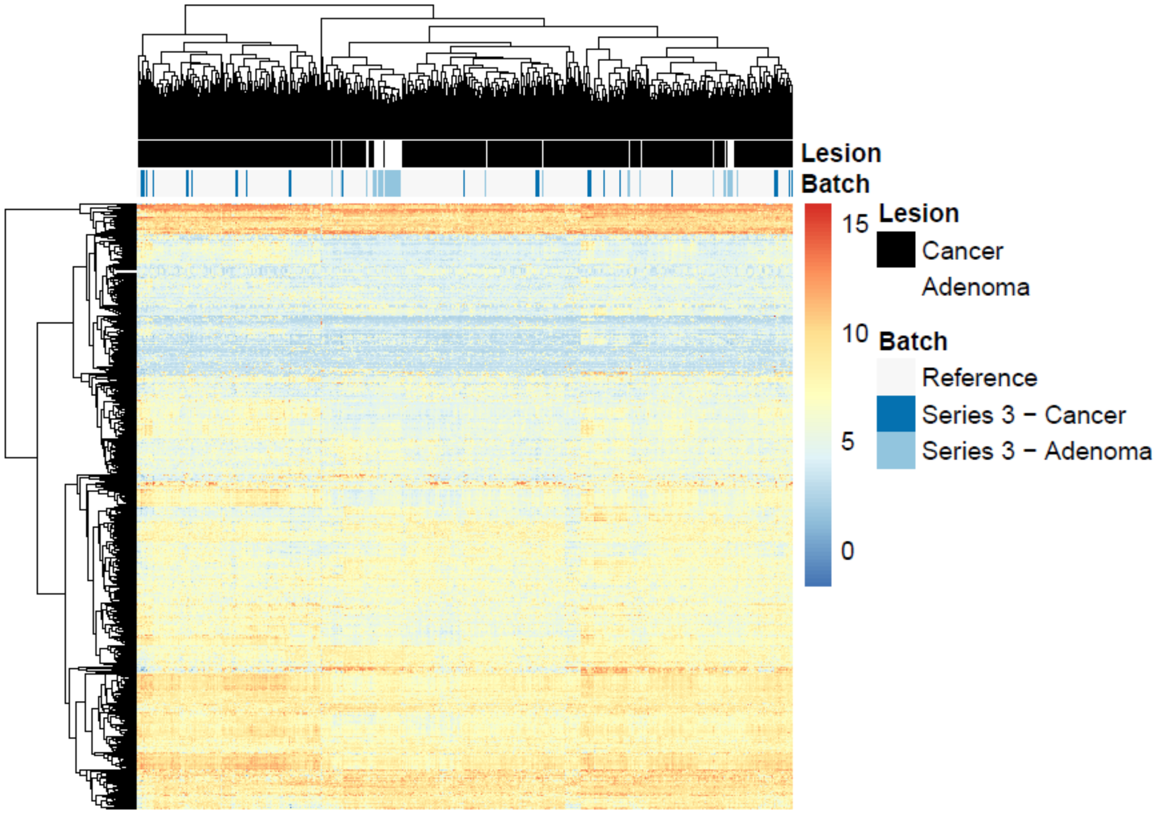
Lesion
Batch
15
10
5
0
Lesion
Cancer
Adenoma
Batch
Reference
Series 3 − Cancer
Series 3 − Adenoma

D

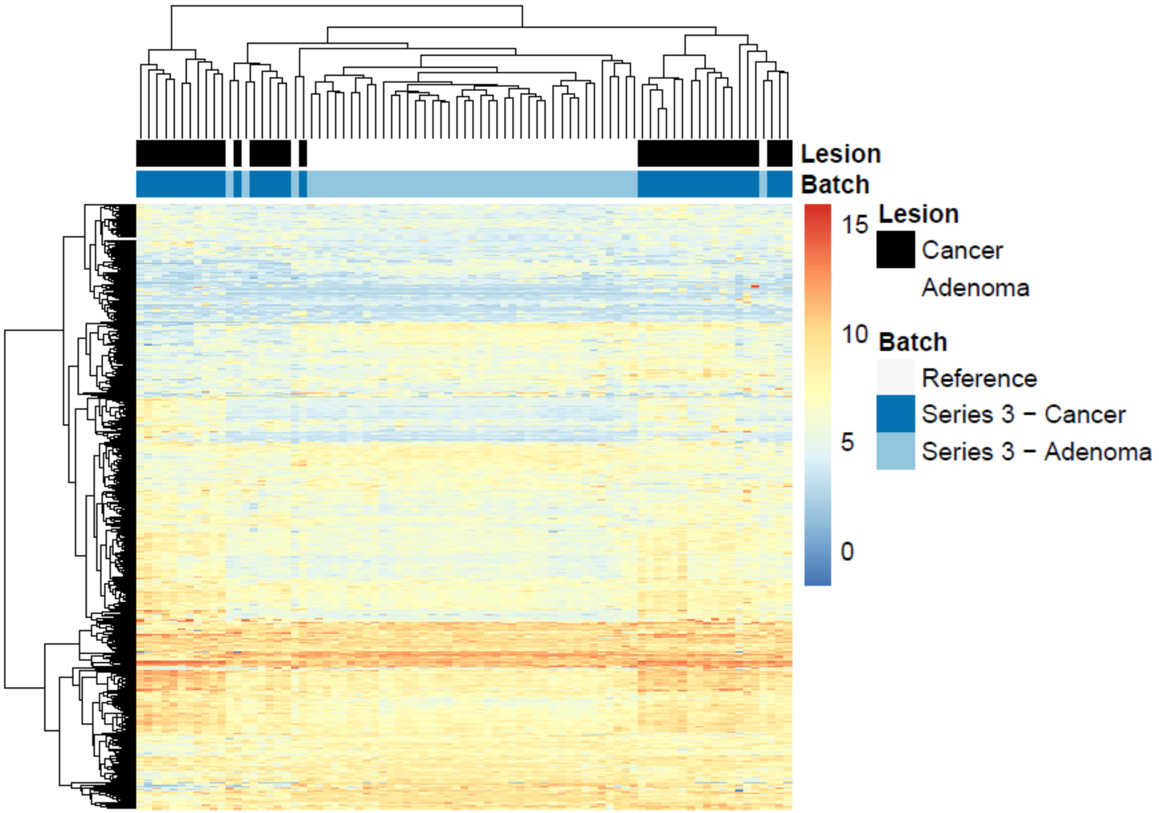
Lesion
Batch
15
10
5
0
Lesion
Cancer
Adenoma
Batch
Reference
Series 3 − Cancer
Series 3 − Adenoma

Figure S4. Hierarchical clustering based on the gene expression profiles of the top 1000 most variable genes. Reference is the reference dataset used for normalisation, Series 3 is the validation set. (A) Heatmap of the two datasets before batch effect removal. The batches corresponding to the Reference and Series 3 can be distinguished in the heatmap. (B) Heatmap before batch correction of the Series 3 only. Clusters enriched with adenomas and clusters enriched with cancers can be distinguished. (C) Heatmap of the two datasets after batch effect removal. Samples from the two experiments do not cluster together. (D) Heatmap of the Series 3 after batch effect removal. Clusters enriched with adenomas or cancers can still be distinguished, meaning that batch effect correction did not remove the variability between different lesions.

Figure S5

A

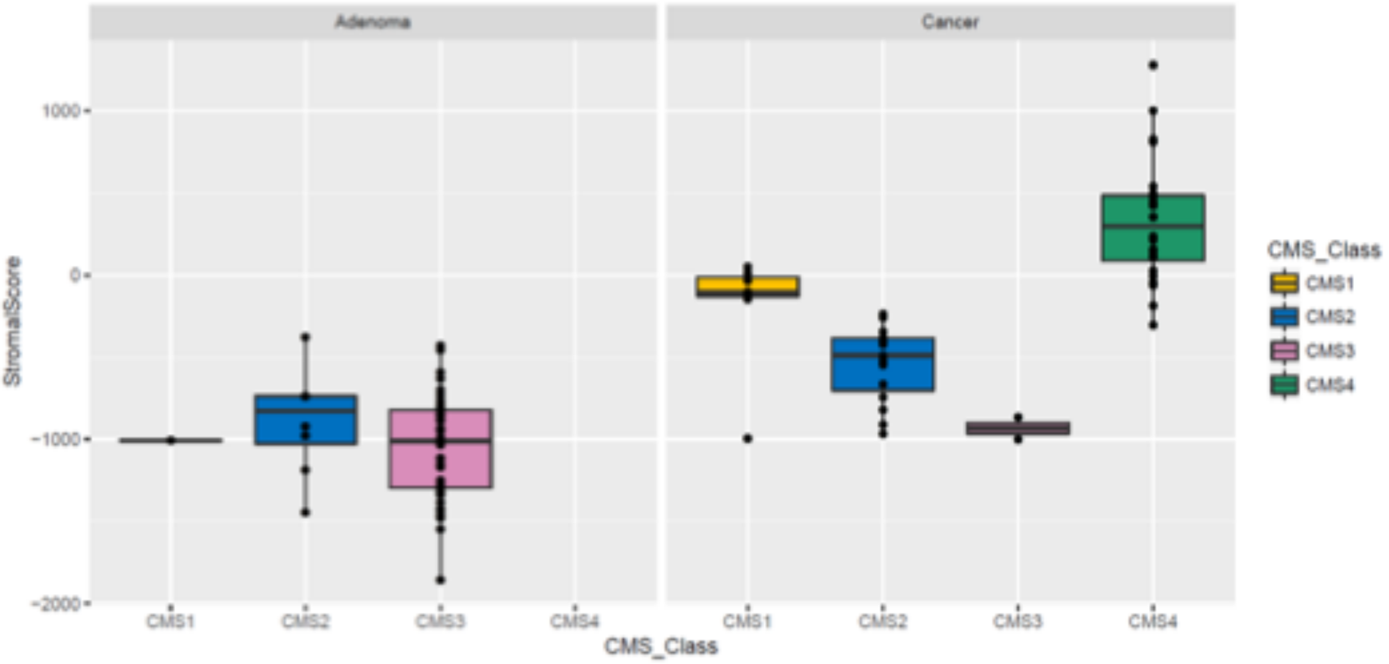
Adenoma
Cancer
StromalScore
1000
0
−1000
−2000
CMS1
CMS2
CMS3
CMS4
CMS_Class
CMS_Class
CMS1
CMS2
CMS3
CMS4

B

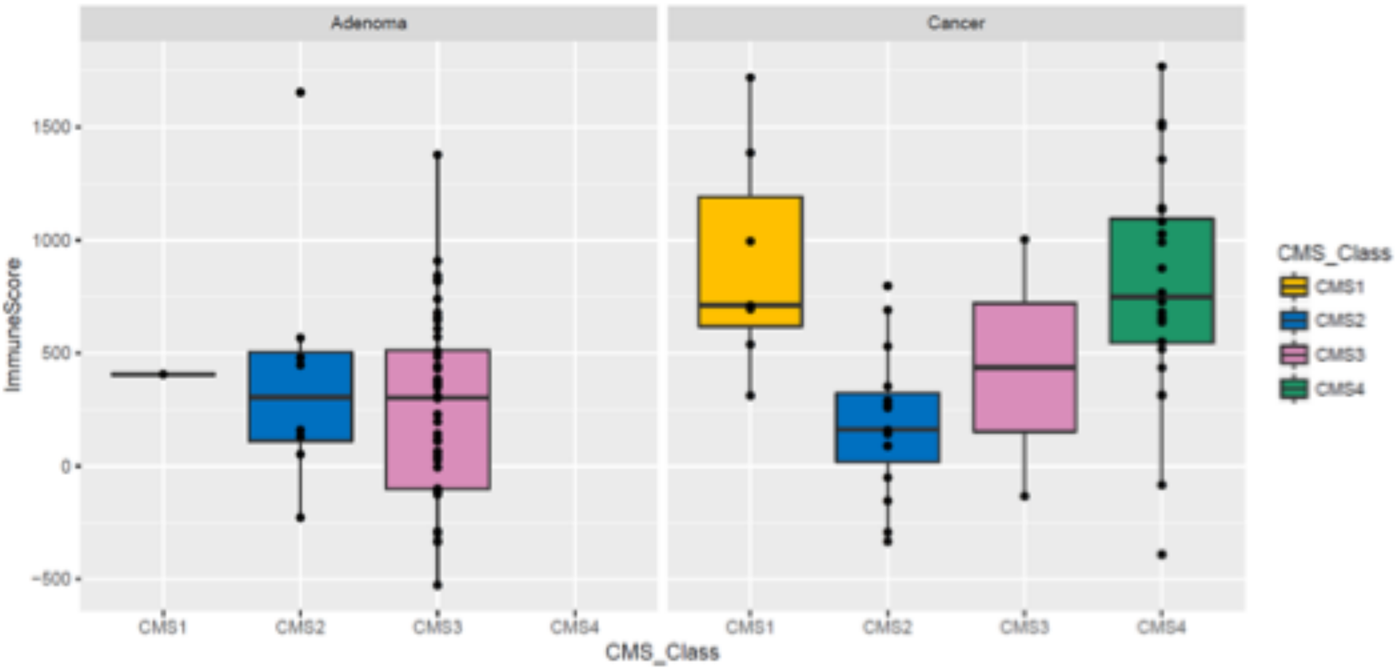
Adenoma
Cancer
ImmuneScore
1500
1000
500
0
−500
CMS1
CMS2
CMS3
CMS4
CMS_Class
CMS_Class
CMS1
CMS2
CMS3
CMS4

Figure S5

C

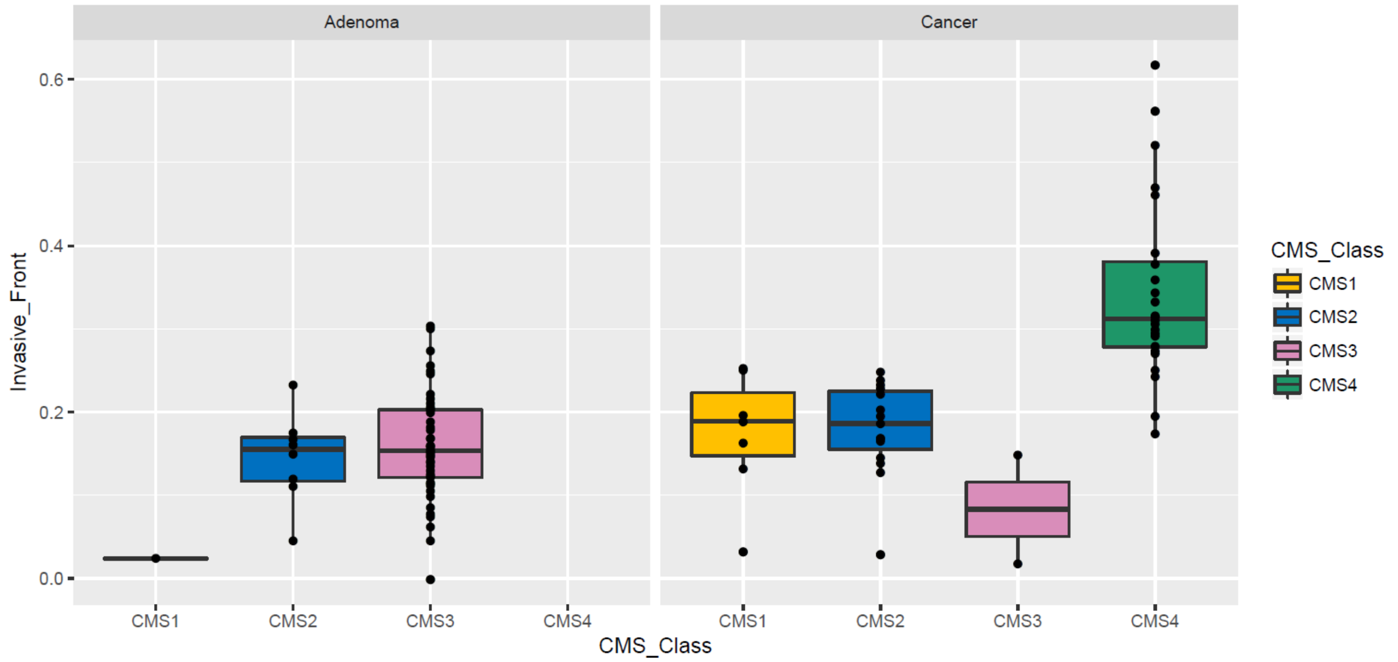

D

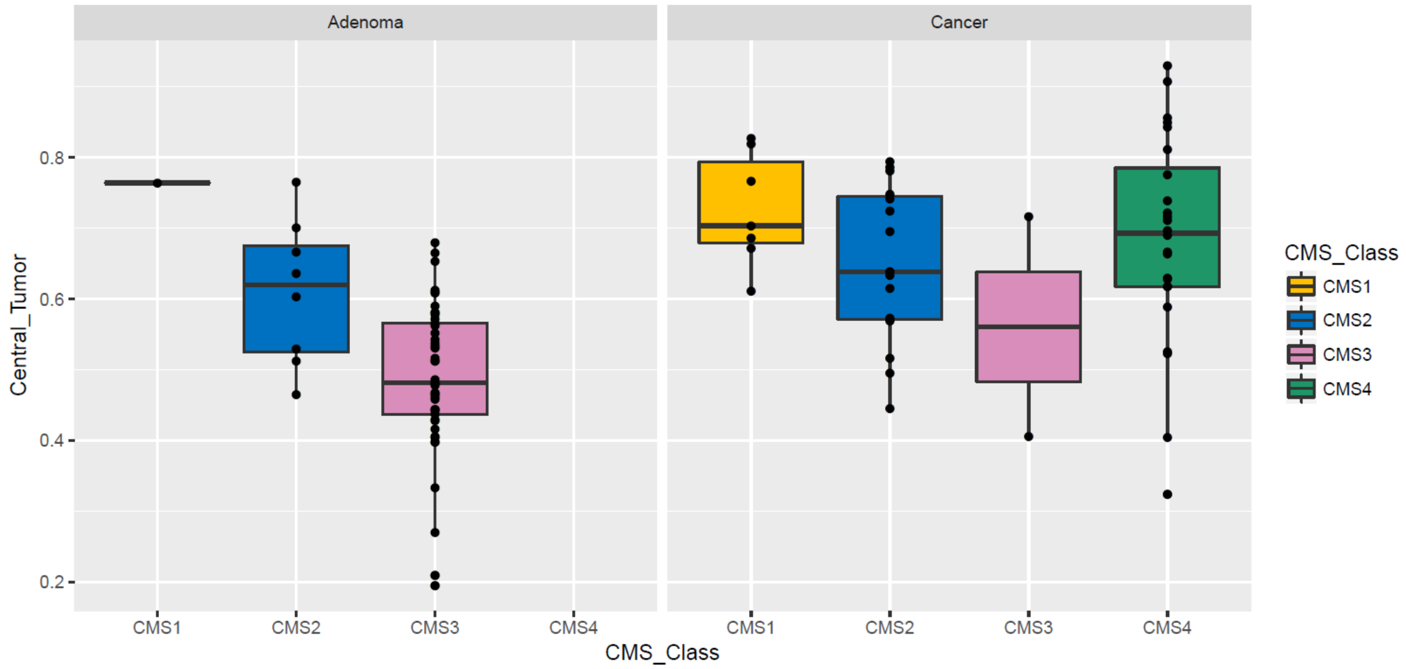

Figure S5. ESTIMATE scores and ssGSEA enrichment scores among CMS classes in adenomas and cancer. “Stromal” (A) and “Immune” (B) scores were calculated using the ESTIMATE algorithm and plotted per CMS group in colorectal adenomas and cancers. “Invasive Front” (C) and “Central Tumor” (D) enrichment was calculated using the ssGSEA algorithm.
